# Rapid genomic sequencing for genetic disease diagnosis and therapy in intensive care units: a review

**DOI:** 10.1038/s41525-024-00404-0

**Published:** 2024-02-27

**Authors:** Stephen F. Kingsmore, Russell Nofsinger, Kasia Ellsworth

**Affiliations:** Rady Children’s Institute for Genomic Medicine, Rady Children’s Hospital, San Diego, CA USA

**Keywords:** Molecular medicine, Genetic testing, Genetic testing

## Abstract

Single locus (Mendelian) diseases are a leading cause of childhood hospitalization, intensive care unit (ICU) admission, mortality, and healthcare cost. Rapid genome sequencing (RGS), ultra-rapid genome sequencing (URGS), and rapid exome sequencing (RES) are diagnostic tests for genetic diseases for ICU patients. In 44 studies of children in ICUs with diseases of unknown etiology, 37% received a genetic diagnosis, 26% had consequent changes in management, and net healthcare costs were reduced by $14,265 per child tested by URGS, RGS, or RES. URGS outperformed RGS and RES with faster time to diagnosis, and higher rate of diagnosis and clinical utility. Diagnostic and clinical outcomes will improve as methods evolve, costs decrease, and testing is implemented within precision medicine delivery systems attuned to ICU needs. URGS, RGS, and RES are currently performed in <5% of the ~200,000 children likely to benefit annually due to lack of payor coverage, inadequate reimbursement, hospital policies, hospitalist unfamiliarity, under-recognition of possible genetic diseases, and current formatting as tests rather than as a rapid precision medicine delivery system. The gap between actual and optimal outcomes in children in ICUs is currently increasing since expanded use of URGS, RGS, and RES lags growth in those likely to benefit through new therapies. There is sufficient evidence to conclude that URGS, RGS, or RES should be considered in all children with diseases of uncertain etiology at ICU admission. Minimally, diagnostic URGS, RGS, or RES should be ordered early during admissions of critically ill infants and children with suspected genetic diseases.

## Introduction

For three thousand years literature has attested to the ideal of an earth without infant mortality or parental grief^1^. However, infant mortality remains unacceptably high in the United States (5.6 deaths per 1000 births in 2022)^2^. Indeed, the US ranked thirty seventh in infant mortality among Organization for Economic Cooperation and Development countries in 2021^3^. While infant mortality was a little lower in San Diego County, California (3.7 deaths per 1000 births in 2022), genome sequencing showed that single locus (Mendelian) genetic diseases were associated with 41% of infant deaths between 2015 and 2022 in that county^4^. Furthermore, 31% of those genetic diseases had effective therapies^4^. Had those genetic diseases been diagnosed at birth and indicated treatments been initiated, infant mortality may have been decreased—by up to 17% in that setting. If the estimate that 5.3% of 3.7 million United States newborns will suffer from a genetic disorder is correct, genome sequencing has the potential to change outcomes of many infants^5,6^.

## Background

**Fig. 1:**
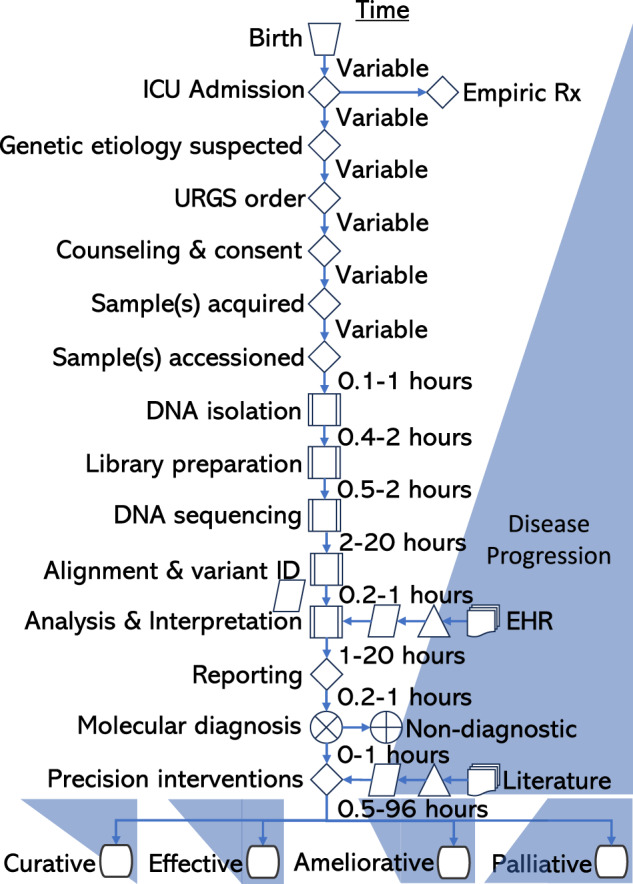
Timeline and process flow graph demonstrating that for clinical URGS to improve outcomes in children admitted to ICUs it must be incorporated within a multi-disciplinary healthcare delivery system. While clinical grade URGS can be in performed in as little as 20 h, it typically takes at least 36 h. However, time-to-diagnosis by URGS is dependent upon four antecedent steps that typically take at least 24 h. Furthermore, improvements in outcome only start to occur upon implementation of precision dietary, drug, device, or surgical interventions. While almost every molecular diagnosis changes management, very few genetic diseases currently have curative treatments. Rx treatment, ID identification, EHR electronic health record. Icons:  manual operation;  decision;  predefined process;  data;  extract;  multidocument;  summing junction; or;  terminator.

First described twelve years ago, ultra-rapid genome sequencing (URGS) is a purpose-developed, clinical method for timely (2 day) diagnosis of genetic diseases in neonatal, pediatric, and cardiovascular intensive care units (NICU, PICU, CVICU)^7^. Since then, URGS has evolved into a scalable delivery system for molecular diagnosis of genetic diseases, metagenomic identification of pathogens, and therapeutic intervention in inpatient children (Fig. 1)^6–15^. Rapid genome and exome sequencing (RGS, RES) are variations of URGS, but with time-to-result of ten to fourteen days and, for the latter, sequencing limited to the ~1% of the genome that is exons of genes. Subsequent studies have shown that URGS, RGS, and RES are effective, universal, agnostic methods for single locus (Mendelian) genetic disease diagnosis [reviewed in 6]. When timely diagnosis is combined with targeted therapeutic interventions, URGS, RGS, and RES are associated with changes in outcome for 18% of NICU and PICU children^6^. Herein we review progress in URGS over the first twelve years and explore prospects for genome-informed healthcare for children with genetic diseases. This review is intended to be complementary to a recent review of the role of genome sequencing in NICUs^6^.

## Diversity of genetic diseases

**Fig. 2:**
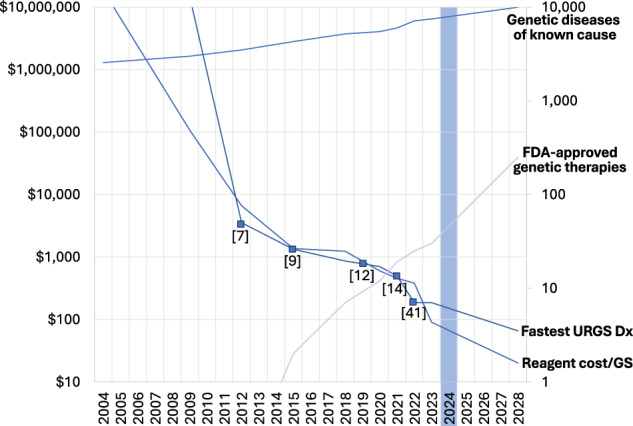
Temporal evolution of genetic disease knowledge and treatment, and genome sequencing since 2004. Fastest time to diagnosis by URGS is in hours. The number of known genetic diseases (light blue) and approved genetic therapies (black) are integers. Reagent cost per GS (genome sequence) is in US dollars. The numbers in parentheses are citations for fastest times to URGS-based genetic disease diagnosis.

**Fig. 3:**
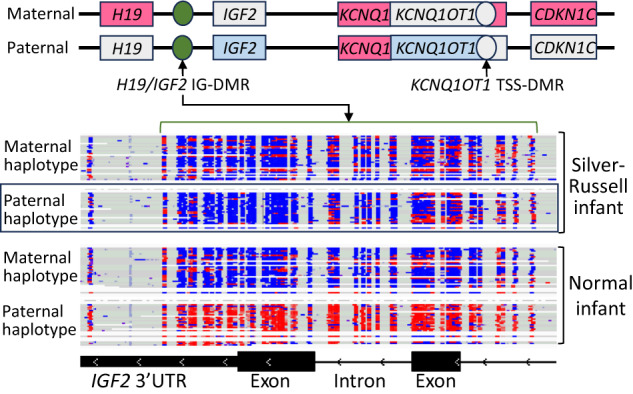
Diagnosis of Silver-Russell syndrome by 5mC detection by LRGS. Top section: Maternal and paternal features of the chromosome 11p15 region. *IGF2*, insulin-like growth factor 2. *H19*, imprinted maternally expressed noncoding transcript. *KCNQ1*, voltage-gated KQT-like potassium channel 1. *CDKN1C*, cyclin-dependent kinase inhibitor 1C. *KCNQ1OT1*, *KCNQ1*-opposite strand antisense transcript 1. TSS-DMR, transcriptional start side differentially methylated region. IG-DMR, intergenic differentially methylated region. Paternal hypomethylation of *H19*/*IGF2* IG-DMR (green, nt 2,132,500-2,134,500) results in loss of paternal *IGF2* expression (light blue) and gain of maternal *H19* expression (pink), which lead to growth restriction. Middle section: Phased, aligned reads of 80X Oxford Nanopore LRGS to Chr 11 nt 2,132,000–2,134,520 in an infant with Silver-Russell Syndrome (above) and a control (below). Reads are shown as individual rows. Individual cytosine nt are highlighted in blue. 5mC are highlighted in red. In the affected infant, the paternal haplotype (black box) shows abnormal hypomethylation (blue) of the *H19*/*IGF2* IG-DMR. In the control, the paternal haplotype shows normal methylation (red) of the H19/IGF2 IG-DMR. Bottom section: *IGF2* introns and exons on the Chr 11 nt 2,132,000–2,134,520.

As of January 2024, Online Mendelian Inheritance in Man (OMIM) listed 7476 single locus (Mendelian) diseases that are causally associated with 4876 genes^16^. This number increases by ~530 disease-gene dyads per year (Fig. 2)^17^. At that time, dbVar listed 27,109 distinct pathogenic or likely pathogenic structural genomic variations (SV, variants of length greater than 50 nucleotides)^18^. This number increases by ~3400 per year. The total number of unique genetic disorders represented by SV is not known. For example, dbVar lists 1088 pathogenic or likely pathogenic SV associated with Duchenne Muscular Dystrophy^19^. However, most ClinVar pathogenic and likely pathogenic SV lack sufficient phenotype information to be mapped to syndromes, precluding enumeration of associated disorders. Furthermore, ClinVar, dbVar, and OMIM overlap by listing classical chromosomal anomalies (Fig. 3).

Strictly speaking, not all of these are single locus disorders since our current representations of SV are simplified. They are informed by technologies such as short read sequencing, chromosomal microarrays, karyotyping, and fluorescent in situ hybridization. Newer technologies, such as long-read sequencing and optical mapping, are demonstrating that many SV are more complex than hitherto recognized^20,21^. Nevertheless, we distinguish these “single event”, uncommon genetic diseases from common, complex disorders which etiologically reflect the aggregate of many common and rare variants together with other risk factors. Furthermore, not all single locus genetic diseases have Mendelian inheritance. The most common occurrence of genetic diseases in the US is de novo – arising either in gametes or early in embryogenesis^22^. Single locus genetic disorders also include other non-Mendelian inheritance mechanisms such as imprinting disorders, mitochondrial genome disorders, retrotransposition, gene conversion events, and repeat expansion disorders^23–25^.

## Estimated incidence of genetic diseases

The aggregate birth incidence and childhood prevalence of single locus genetic diseases is not definitively known. Verma and Puri suggest the aggregate incidence to be 5.3%^5^. They note that the aggregate incidence varies by race, ethnicity, and geography^5^. The incidence of recessive disorders is higher in cultures with frequent consanguineous marriages^26^. Two of the most common types of genetic disease, hemoglobinopathies and glucose-6-phosphate dehydrogenase deficiency, have a higher incidence in individuals whose ancestry is from regions affected in the past by severe falciparum malaria^26,27^. An alternative method for estimating the minimal prevalence of genetic diseases is the prevalence of clinically actionable secondary findings of genome or exome sequencing using the American College of Medical Genetics recommended lists. These range between 2% and 3%, albeit these prevalence estimates will increase as the recommended secondary findings list expands over time^28–31^. Data from two clinical studies suggests the incidence of single locus genetic diseases in regional NICUs to be ~15%^32,33^. Healthcare costs of children with genetic diseases are ~5 fold higher than other chronic diseases^34^. There are ~400,000 US NICU admissions per annum, costing over $17 billion per annum^35–37^. By extrapolation from California Department of Health statistics, there are ~200,000 US PICU admissions per annum. The incidence of genetic diseases in NICU, PICU and CVICU admissions is almost certainly higher than current estimates, since almost all published studies of URGS, RGS and RES have been limited to infants and children with suspected genetic diseases and to phenotype-related diagnostic findings. Further studies are needed to understand the true incidence of genetic diseases – which will inform the optimal scope of testing – particularly in NICU, PICU and CVICU patients who are not currently suspected of having genetic diseases.

## Diagnostic ultra-rapid genome sequencing

Here we define diagnostic URGS and RGS as clinical-grade methods that identify all major types of DNA variation in at least 90% of the nucleotides of an affected patient with at least 95% accuracy. URGS corresponds to methods that consistently return a diagnostic result within 3 days of sample receipt, and RGS and RES within 10 days. RES differs in that DNA variation identification is limited to exons and nucleotides near exon-intron boundaries, leading to a slightly lower diagnostic yield^38,39^. The introduction of new sequencing and informatic technologies has enabled the speed record for URGS to decrease with time, from 48 h in 2012 to 7 h in 2022, albeit such records featured research rather than clinical methods (Fig. 2)^7,9,12,14,40–42^. URGS has very recently become technically possible in 90 min, albeit limited to tumor classification^43^. The median time for return of a provisional diagnostic result of URGS in routine clinical operation is tracked monthly at our Institute, and averages ~36 h.

## URGS methods

Clinical diagnostic URGS and RGS currently have seven steps^12,14^. First, high molecular weight genomic DNA is isolated from proband and parental samples, if the latter are available and provide consent. While many sample types are amenable to URGS, blood and dried blood spots are the two most common types. Genomic DNA includes both the nuclear genome, mitochondrial genome, and – if the newborn has suspected sepsis – pathogen genomes. The second step is conversion of genomic DNA into a format compatible with sequencing (library preparation). This involves random fragmentation of DNA, end-repair, and ligation of adapter sequences. In URGS these steps may be combined and take about 1 h^14^. Third, next-generation sequencing is performed. Currently most URGS uses Illumina “short read” (SR) sequencing-by-synthesis (SBS, Illumina, 11–24 h) although nanopore “long read” (LR) strand sequencing (Oxford Nanopore) is starting to be used because it enables real-time sequence analysis, generates much longer contiguous haplotypes, and detects 5-methyl cytosine (“methylation status”, which is relevant for diagnosis of imprinting disorders or detection of specific DNA methylation episignatures of genetic diseases)^12,14,40–46^. Fourth the sequence fragments (reads) are mapped to a reference human genome and ~5 million variants are identified and genotyped (30 min). For metagenomic pathogen detection, reads are also mapped to a collection of pathogen genomes^13,47,48^. Fifth, each variant is annotated with results of a batch of over twenty automated software tools and variants (or variant diplotypes) are rank ordered in terms of predicted pathogenicity (20 min)^12,14^. Sixth, the phenotypes observed in the affected child are matched to those of all known genetic diseases and a comprehensive, rank ordered differential diagnosis is determined^12,14^. Seventh, the results are interpreted according to guidelines developed by professional societies (such as the American College of Medical Genetics and Genomics, ACMG). This is performed manually by experts (up to 100 variants per case, 10 h), with artificial intelligence (no variant limit, 40 min), or with both. When available, trio evaluation enables facile identification of variants that occurred de novo and, in recessive disorders, whether heterozygous variants are *in cis* or in trans in probands which results in a slightly higher diagnostic yield when trios are available^49^.

Effective use of URGS, RGS, and RES in critically ill children requires numerous pre-test and post-test steps, leading to the use of the term Rapid Precision Medicine to describe the healthcare delivery system of which URGS, RGS, or RES are a part^6,14^.

## Diagnostic utility of URGS

Since the initial description of URGS in 2012, more than 44 studies of the diagnostic utility of URGS, RGS, or RES have been published (Table 1)^7,12,31,32,38,39,48,50–85^. These studies were performed in 12 countries and in 3609 infants and children. The enrollment criteria for these studies varied (Table 1). Almost all were critically ill inpatient children. Most were NICU infants or older PICU children. Almost all were suspected of having genetic diseases or had diseases of unknown etiology at time of test order. A suspected genetic disease is defined as symptoms, signs, or abnormal laboratory tests observed in an affected individual that matches one or more genetic diseases. A disease of unknown etiology is defined as symptoms, signs, or abnormal laboratory tests observed in an affected individual for which the root cause, such as a molecular diagnosis, is unknown. The definition of genetic disease diagnosis was almost always in agreement with professional society guidelines, although a few studies included suspicious variants of uncertain significance (those for which the associated disorder was a good fit with the child’s observed phenotypes)^72,84^. The weighted average diagnostic rate was 37% (range 19%–83%, Table 1). The study designs varied widely. Most were prospective research cohort studies. One study aligned RGS reads to reference pathogen genomes as well as the reference human genome, enabling metagenomic diagnosis of infectious diseases^38^. As has been observed in case reports, pathogens were identified in 6 (3%) of 202 infants, each of whom had symptoms of sepsis, and guided antibiotic treatment^13,38,47^. Since sepsis is suspected in many NICU infants at time of admission, the addition of this simple additional bioinformatic step is likely to become generally adopted.

**Table 1:** Studies of the diagnostic performance, change in management and outcome, and time to result (TAT) of URGS, RGS and RES in seriously ill children in intensive care units

Ref.	Year	Country	Study Type	Test	Enrollment Criteria	Study size	Dx Rate	Δ Mx	Δ Outcome	TAT (days)
^7^	2012	USA	Cases	URGS	NICU infants; Susp. genetic dis.	4	75%	n.d.	n.d.	2
^48^	2015	USA	Cohort	RGS	<4 months of age; Susp. actionable genetic dis.	35	57%	31%	29%	23
^50^	2017	USA	Cohort	RES	<100 days old; Susp. genetic dis.	63	51%	37%	19%	13
^51^	2017	Holland	Cohort	RGS	Infants; NICU, PICU; Susp. genetic dis.	23	30%	22%	22%	12
^52^	2018	USA	RCT	RGS, SOC	<4 months of age; Susp. genetic dis.	32	41%	31%	n.d.	13
^53^	2018	USA	Cohort	RGS	Infants; Susp. genetic dis.	42	43%	31%	26%	23
^54^	2018	Aust	Cohort	RES	Acutely ill children with susp. genetic dis.	40	53%	30%	8%	16
^55^	2018	UK	Cohort	RGS	Children; PICU and Cardiovascular ICU	24	42%	13%	n.d.	9
^56^	2019	USA	Cohort	RGS	4 months-18 years; PICU; Susp. genetic dis.	38	48%	39%	8%	14
^57^	2019	UK	Cohort	RGS	Susp. genetic dis.	195	21%	13%	n.d.	21
^12^	2019	USA	Cases	URGS	Infants; ICU; Susp. genetic dis.	7	43%	43%	n.d.	0.8
^58^	2020	USA	Cohort	RES	<6 months old; ICU; hypotonia, seizures, metabolic, multiple congenital anomalies	50	58%	48%	n.d.	5
^59^	2019	Canada	Cohort	RES	NICU; infants; susp. genetic dis.	25	72%	60%	n.d.	7.2
^60^	2019	Taiwan	Cohort	RES	PICU and other; children; susp. genetic dis.	40	53%	43%	n.d.	6
^61^	2020	China	Cohort	RES	NICU & PICU; complex	130	48%	23%	n.d.	3.8
^62^	2020	USA	Cohort	RES	Critical illness; medical genetics selected	46	43%	52%	n.d.	9
^63^	2020	USA	Cohort	RES	PICU; < 6 years; new metabolic/neurologic dis.	10	50%	30%	n.d.	9.8
^64^	2020	USA	Cohort	RES	ICU; infants	368	27%	n.d.	n.d.	n.d.
^65^	2020	China	Cohort	RES	Infants; ICU and inpatient	102	31%	27%	n.d.	11
^66^	2020	USA	Cohort	RES	Various	41	32%	n.d.	n.d.	7
^67^	2020	Aust	Implem	URES	<18 year; NICU and PICU	108	51%	44%	n.d.	3
^68^	2020	Poland	Cohort	RES	Infants; NICU, PICU; susp. genetic dis.	18	83%	61%	n.d.	14
^69^	2020	China	Cohort	URES	Infants; NICU, PICU; susp. genetic dis.	33	70%	30%	30%	1
^32,70,71,169^	2019, 2020,2023	USA	RCT	RGS	Infants; dis. of unknown etiology; within 96 h of admission	94	19%	24%	10%	11
^32,70,71,169^	2019, 2020,2023	USA	RCT	RES	Infants; dis. of unknown etiology; within 96 h of admission	95	20%	20%	18%	11
^32,70,71,169^	2019, 2020,2023	USA	RCT	URGS	Infants; dis. of unknown etiology; within 96 h of admission	24	46%	63%	25%	4.6
^73^	2021	USA	Implem	URGS	Medicaid infants; unknown etiology; within 1 week of admission	184	40%	32%	n.d.	3
^74^	2021	China	Cohort	RES	Critically ill; 6 days - 15 years; susp. genetic dis.	40	43%	31%	n.d.	5
^75^	2021	Germany	Cohort	RES	NICU, PICU, infants; sup. genetic dis.	61	43%	11%	n.d.	60
^76^	2021	USA	RTDCT	RGS, WGS	<120 days old; ICU; susp. genetic dis.	354	31%	25%	n.d.	15
^38^	2021	China	Crossover	RES	Critically ill infants with susp. genetic heterogeneous dis.	202	20%	n.d.	n.d.	20
^38^	2021	China	Crossover	RGS	Critically ill infants with susp. genetic heterogeneous disorders	202	37%	7%	n.d.	7
^77^	2022	France	Cohort	RGS	Critically ill infants with susp. genetic heterogeneous disorders	37	57%	n.d.	n.d.	43
^78^	2022	UAE	Cohort	URGS	Infants in ICU with complex multisystem dis.	5	60%	20%	20%	1.5
^79^	2022	USA	Implem	RES	NICU infants with susp. genetic dis.	80	28%	18%	n.d.	13
^80^	2022	USA	Cohort	RGS	Children in ICU with dis. of unknown etiology	65	40%	n.d.	n.d.	12
^81^	2022	France	Cohort	RES	Infants in ICU with susp. genetic dis.	15	40%	53%	n.d.	16
^82^	2023	USA	Implem	RGS	NICU, PICU with dis. of unknown etiology	89	39%	27%	n.d.	n.d.
^72,83^	2021, 2023	USA	Crossover	RGS, panel	NICU with dis. of unknown etiology	400	49%	19%	n.d.	6
^84^	2023	USA	Cohort	RGS	Acutely ill inpatient infants; susp. genetic dis.	188	35%	32%	n.d.	6
^85^	2023	Belgium	Cohort	URGS	NICU, PICU, neurologic inpatients with susp. genetic dis.	21	57%	57%	n.d.	1
Weighted Average						3609	37%	26%	18%	n.d.

Study size refers to the number of probands. Studies are listed from oldest to newest.

*Ref.* reference, *Δ* Change, *Dx* diagnosis, *Mx* management, *TAT* turnaround time, *n.d.* not done, *d* days, *RCT* Randomized Controlled Trial, *SOC* standard of care, *Implem* implementation science design, *RTDCT* randomized time delayed clinical trial, *Crossover* Patients received both interventions, *Dis.* disease, *Susp.* suspected, *Aust* Australia, *UAE* United Arab Emirates.

Two studies were randomized controlled trials (RCT)^32,52,70,71^. The Newborn Sequencing in Genomic Medicine and Public Health (NSIGHT) RCT compared rapid genome sequencing (RGS) with standard of care testing in NICU and PICU infants aged <4 months with suspected genetic diseases^52^. The primary endpoint (rate of genetic diagnosis within 28 days of enrollment) was met, with 31% in RGS group and 3% in controls. The median age at diagnosis was less in the RGS group (25 days) than in controls (130 days), and median time to diagnosis was less in the RGS group (13 days) than in controls (107 days). The study was terminated early due to loss of equipoise. A meta-analysis published in 2018 reported diagnostic genome sequencing [(41% diagnosis) and exome sequencing (36% diagnosis) to be superior to chromosomal microarrays (10% diagnosis) 49]. Meta-analysis also showed that the likelihood of diagnosis was significantly greater for trio testing than singletons (odds ratio 2.04)^49^. A second meta-analysis of the utility of RGS and RES in 2022 reported diagnostic rates of 37% and 50%, respectively^86^.

The NSIGHT2 RCT compared RGS with RES in infants in ICUs with disorders of unknown etiology^32,70,71^. Forty six percent of NICU admissions were eligible for enrollment in NSIGHT2, a much greater proportion than in other studies. As a result, however, the diagnostic rate was less ( ~ 20%) and the study was underpowered to test the hypothesis that RGS was associated with a higher rate of genetic disease diagnosis than RES.

Two studies had crossover designs in which all enrolled infants received two interventions. In GEMINI, infants received both RGS and a rapid gene panel test^72,83^. In the China Neonatal Genomes Project, infants received both RGS and RES tests^38^. RGS demonstrated superiority in both studies. The diagnostic yield of RGS in GEMINI was 49%, compared with 27% with the rapid gene panel test^83^. In the Chinese study, the diagnostic yield of trio-RGS was higher than that of proband-only clinical RES (36.6% vs 20.3%, respectively), and the median turnaround time for trio-RGS (7 days) was faster than that of proband-only clinical RES (20 days)^38^.

With the cutoff of 3-day time to result, only seven studies evaluated the diagnostic utility of URGS^7,12,67,69,73,78,85^. In 356 children it was 48%, which was higher than the remaining studies of RGS or RES (36%). There are two likely reasons for this difference. Firstly, URGS is reserved for infants and children with the most severe illnesses and in whom there is greater likelihood that results will inform substantial changes in care – such as severe metabolic derangement, cardiac dysrhythmias, hypotonia, and drug-resistant seizures – which may have higher diagnostic yield. Secondly, URGS is more likely to be used as a first-tier test which also increases diagnostic yield compared with use after, for example, a negative chromosomal microarray or gene panel test.

## Clinical utility of URGS

Thirty nine of the 44 studies of the diagnostic utility of RGS, URGS, or RES also evaluated the proportion of children who received changes in management upon return of results (Table 1)^12,32,38,48,50–63,65,67–76,78,79,81–85^. Definitions of changes in management varied between studies, but were usually changes in surgical, dietary, drug, and device interventions. Inclusion of changes in diagnostic testing and subspeciality consultation were variable. Changes in genetic counseling were not included. In 2,858 infants and children the weighted average rate of change in management was 26% (range 7% − 63%). With the cutoff of 3-day time to result, six studies evaluated the clinical utility of URGS (Table 1). In 352 children it was 37%, which was higher than the remaining studies of RGS or RES (25%). This was in accord with the reasoning mentioned above for a higher diagnostic rate for URGS. In addition, it is particularly impactful in terms of clinical management to have results returned to the ordering physician prior to patient discharge as discharge prior to diagnosis will result in some patients lost to follow-up and all patients’ future clinical engagements to be delayed.

URGS is predicated on the assumption that the clinical utility of a genetic disease diagnosis during an ICU admission is inversely proportional to the time to result. URGS is associated with a greater proportion of results returned before NICU discharge or death than RES or RGS^32^. Sixty days after enrollment, one study found that twice as many infants who received clinical genome sequencing results in 15 days (early) had changes in management relative to those who received results in 60 days^76^.

Several studies measured rates of change in management following negative results, in addition to positive results. NSIGHT2 found that clinicians perceived negative results to be useful or very useful in 72% of cases (versus 93% with positive results)^70,71^. That study also found that negative tests changed management in 16% of cases (versus 63% with positive results). The Australian Genomics Acute Care (AGAC) study reported that 11% of negative results influenced clinical management (versus 76% with positive results)^67^. Why negative results change management requires an explanation. Children with a suspected genetic disease may have had additional tests ordered or may have had decisions with regard to interventions that are impacted by that disease. While a negative result cannot completely rule out a genetic disorder it does greatly reduce its likelihood. A negative comprehensive genetic test, like URGS, decreases the likelihood of having any genetic disorder that is within the differential diagnosis. An example from our experience was a child with end-stage cardiomyopathy in whom a negative URGS led to listing for heart transplant. Prior to testing there was concern that a genetic cause of heart failure may have contraindicated transplant. Furthermore, Reanalysis of URGS, RGS, and RES is starting to occur commonly in patients with negative results or with findings that do not fully explain a child’s illness. Reanalysis is typically performed upon the appearance of new disease features or when sufficient time has elapsed for enhancements in diagnostic sensitivity to have occurred. The incremental yield of reanalysis is 1-15% and annual reanalysis has been suggested as a possible best practice in consenting patients^87–90^.

Three studies reported implementation research. AGAC reported implementation of ultra-RES (3-day time to results) in 108 critically ill children with suspected monogenic conditions in 12 hospitals^67^. The diagnostic rate was 51% and management was influenced by results in 44%. Project Baby Bear was a payor funded, prospective, real-world, quality improvement project of URGS in the regional ICUs of five California tertiary care children’s hospitals^73^. The participants were 184 acutely ill Medicaid beneficiaries aged <1 year and within one week of hospitalization, or who had just developed an abnormal response to therapy. Most infants were from underserved populations. URGS (3-day time to results) met two prespecified primary outcomes—changes in medical care reported by physicians and changes in the cost of care. A molecular diagnosis was made in 40% of infants and results led to changes in care in 32%^73^. Project Baby Deer evaluated implementation of URGS in 89 children in NICUs and PICUs of five community hospitals and two children’s hospitals in Michigan^82^. The rate of diagnosis was 39% and results changed management in 27%. Further studies are needed to determine how clinical utility of URGS, RGS and RES vary with time from admission to sample receipt, with rapid versus ultra-rapid testing, and by presentation, disease severity, and rate of progression. Addressing these gaps would inform implementation.

## Cost and cost effectiveness of URGS

While the cost of research grade genome sequencing consumables at highest batching and throughput has declined to as little as $200 per 30-fold genome sequence (Fig. 2), the cost of diagnostic URGS and RGS has decreased only threefold in that period (to ~$7000 for a singleton, 3-day clinical test). As of December 2023, the cost of URGS consumables remains ~$1800 per genome because they are optimized for speed. The consumables used for RGS are less expensive (~$750 per sample) but have longer run time and require sample batching. A delay of several days to accrue a batch of samples is incompatible with URGS. Consumable cost of URGS and RGS are about to change since multiple new sequencing platforms were introduced in 2022–2024 that are less costly, optimized for speed, and do not require batching delays. Expert manpower is the largest component of current URGS, RGS, and RES cost, particularly for bioinformatics, software engineering, interpretation, reporting, quality assurance and regulatory compliance. The cost of interpretation does not differ for singleton and trio testing, nor for URGS and RGS. Obviously, consumable cost is three-fold higher for trio than singleton testing. Software and computation cost for URGS is ~$500 per family. Diagnostic URGS and RGS are currently performed at low volumes (less than 1000 families per year per center). As a result, they have not yet benefitted from scaling efficiencies. The falling cost of consumables, gradual replacement of manual interpretation with artificial intelligence, and scaling efficiencies following from broader adoption in light of reimbursement by payors will decrease the cost of URGS and RGS^12,14,91,92^. However, broad use of artificial intelligence and ascent of the adoption curve will likely take 3 years, at which time a cost of diagnostic URGS–as currently performed – of ~$2500 is likely. As noted below, however, there is much potential for technological improvement in URGS that will both lead to increased diagnostic yield and rate of change in management and to cost.

Ten studies have evaluated the cost effectiveness of URGS and RGS, of which nine with similar methodology are shown in Table 2^6,53,73,80,82,83,93–95^. Based on a median cost per proband tested of $9239 (range $6300–$16,063), the cost per diagnosis was $23,602 (median, range $14,082–$37,480). Assuming the current cost of URGS test ($7,000), the cost per diagnosis would be $17,500. The final study, which used different methods based on population estimates, found a similar cost per diagnosis of $24,873 (based on an RGS cost of $12,188)^96^. The cost per diagnosis was also evaluated by two control methods (Table 2). It was $14,515 per diagnosis by chromosomal microarray and karyotyping ($1888 cost per proband) and $24,074 per diagnosis by a next-generation sequencing-based panel test ($6,500 per proband)^80,83,97^. It should be noted that while chromosomal microarray and karyotyping are less expensive per diagnosis than URGS or RGS, the associated rate of change in management was one fifth that of URGS or RGS (Table 2).

**Table 2:** Studies of the cost effectiveness of URGS and RGS in seriously ill children in intensive care units

Ref.	Year	Country	Number of probands	Dx rate	Δ Mx	RGS cost per proband	Cost per Dx	TAT	Net savings per proband
^53^	2018	USA	42	43%	33%	$16,063	$37,480	23	$18,741
^73^	2021	USA	184	40%	32%	$9239	$23,602	3	$6294
^6^	2022	USA	61	33%	n.d.	$9758	$29,570	n.d.	$11,286
^93^	2022	USA	38	45%	34%	$6300	$14,082	14	($1436)
^80^	2022	USA	65	40%	n.d.	$11,029	$27,573	12	$100,440
^94^	2022	Australia	40	53%	39%	$8088	$15,406	3	$17,243
^82^	2023	USA	89	39%	27%	$7564	$19,395	n.d.	$4155
^95^	2023	USA	184	40%	32%	$14,450	$36,125	3	$22,395
^83^	2023	USA	400	49%	n.d.	$8000	$16,326	6	n.d.
Median				40%	33%	$9239	$25,588	$6	$14,265
Controls									
^80,97^	2011, 2022	USA	2098	13%	6%	$1887	$14,515	n.d.	n.d.
^72,83^	2021, 2023	USA	400	27%	n.d.	$6500	$24,074	4	n.d.

Control study 75 featured 305 infants who received standard of care genetic testing for suspected genetic diseases (57% received chromosomal microarray ($1500), karyotype ($600) and newborn screening ($210), 31% received chromosomal microarray and newborn screening, and 1% received next generation sequencing based gene panels). Control study 78 featured 400 infants who received Quest NewbornDx, a next generation sequencing based gene panel.

*Δ* change, *Dx* diagnosis, *Mx* management, *n.d.* not done, *TAT* turnaround time.

Eight of the nine studies used specific disease case-based modeling of actual cost versus counterfactual cost^6,53,73,80,82,93–95^. The median cost savings per child tested, net of test cost, was $14,265. One of these studies compared cost in a cohort receiving RGS with a cohort receiving chromosomal microarray and karyotyping, was an outlier with $100,440 net savings per child tested^80^. Assuming the current cost of URGS test ($7000), the median cost savings per child tested was $16,504. Two of the studies were implementation research undertaken to inform policy^73,82^. Project Baby Bear, discussed above, found that URGS in 184 Medicaid infants in California Children’s Services accredited level four NICUs was associated with $6294 net savings per child tested^73^. Project Baby Deer, also discussed above, found that RGS in in NICUs and PICUs of five community hospitals and two children’s hospitals in Michigan was associated with $4,155 savings per child tested^82^. Three studies that evaluated URGS found median net cost savings of $17,243 per child tested^73,94,95^. Given the increasing per diem costs of care in NICUs and PICUs, it is likely that current cost savings per child tested are greater than these estimates.

As noted above, the clinical utility of a genetic disease diagnosis during an ICU admission has been reported to be inversely proportional to the time to result^32,76^. Three studies modeled the impact of time to result of URGS on cost effectiveness^6,73,95^. Maximal cost savings were apparent with 3-day time to result. Cost savings were less at 7-days, and less again with 14-day time to result. Efficacy of URGS or RGS varies with time from admission to test order and from order to reporting as well as by presentation, disease severity, and rate of progression. Time to result is inversely proportional to test cost, while length of an ICU stay is directly proportional to healthcare expenditures. As supported by the findings of the NSIGHT RCT (32) and Project Baby Bear (73), it is reasonable to expect more rapid diagnostic results to have higher impact on improving both clinical outcomes while simultaneously reducing overall healthcare costs. Accordingly, further studies are needed to define the ideal cost:speed ratio of URGS in high acuity patients to optimize for both outcome improvements and healthcare expenditures.

## Guidelines for clinical use of URGS in the molecular diagnosis of genetic diseases

Diagnostic URGS is performed in the US in genetic laboratories accredited under the Clinical Laboratory Improvement Amendments (CLIA, 1988) and by the College of American Pathologists (CAP) and individual states. CAP performs proficiency testing of laboratories performing URGS. No US Food and Drug Administration-approved tests for RGS or URGS are available currently. CAP, ACMG and the Association for Molecular Pathology (AMP) have issued detailed guidance to diagnostic laboratories regarding genome sequencing, bioinformatics, and interpretation and reporting of single and polynucleotide substitution and insertions and deletion variants, copy number variants, and structural variants identified by genome or exome sequencing^98–102^.

The ACMG issued a 2021 practice guideline in 2021 recommending first- or second-tier diagnostic genome or exome sequencing for all individuals with congenital anomalies, developmental delay, or intellectual disability^103^. The National Society of Genetic Counselors issued a practice guideline in 2022, recommending first-tier diagnostic genome or exome sequencing for all individuals with unexplained epilepsy^104^. As yet there are no professional society practice guidelines for URGS, RGS, or RES for inpatient children.

## URGS coverage policies and test reimbursement

The United Kingdom (UK) released a coverage policy for rapid diagnostic genome sequencing in all inpatient children with suspected genetic diseases in 2019^6^. Pediatricians in the National Health Service (NHS) in England and Wales can order a rapid diagnostic genome for any child that they suspect may benefit. The NHS plans to supplement this with URGS in selected children.

Blue Shield of California was the initial US payer with a coverage policy and reimbursement rate for URGS and RGS in NICU and PICU children in 2019^105^. It has since been endorsed by the Blue Cross Blue Shield Association and adopted by plans federally and in Hawaii, Louisiana, Michigan, Mississippi, Western and Northeastern New York, Idaho, Florida, and New Jersey^106–114^. Recently United Health Group and Cigna have issued coverage policies for RGS, but their application in the inpatient setting has not yet been demonstrated^115,116^.

In 2021, Michigan was the initial state with a Medicaid coverage policy and reimbursement rate for inpatient RGS separate from the Diagnosis Related Group (DRG) payment using Current Procedural Terminology (CPT) codes [81,425 and 81,426 ($4166 singleton, $8652 trio) and 0094U ($6278 singleton, $10,764 trio) 117,118]. In large measure this policy was issued in response to the results of the previously described project Baby Deer^82^. In 2022 − 2024, Louisiana, Minnesota, Florida, Arizona, Utah, and Georgia issued similar coverage policies and reimbursement^117,118^. In 2022, California, Oregon, and Maryland issued similar coverage policies but without reimbursement separate from the DRG^119–121^. California’s policy followed legislative action in response to the results of project Baby Bear^73^. Since DRG-related Medicaid payments to hospitals generally cover 90% − 93% of costs, the absence of a payment apart from the DRG in California has not led to an increase in clinical use of URGS and RGS^122^.

These coverage policies have identified the indications for URGS and RGS in inpatient children. The policies differ regarding whether infants or all children are covered beneficiaries and whether they must be in a NICU, PICU, or inpatient. In general, however, they are aligned. The Michigan Medicaid policy states that RWGS (or URGS) is medically necessary when all the following apply^123,124^:

1. Signs/symptoms suggest a rare genetic condition that cannot be diagnosed by a standard clinical work-up;
2. The beneficiary’s signs/symptoms suggest a broad, differential diagnosis that could require multiple genetic tests if RGS was not performed;
3. Timely identification of a molecular diagnosis is necessary to guide clinical decision making, and the RGS results will guide the treatment and/or management of the beneficiary’s condition; and
4. At least one of the following clinical criteria apply:
  - Multiple congenital anomalies,
  - Specific malformations highly suggestive of a genetic etiology,
  - An abnormal laboratory test suggests the presence of a genetic disease or complex metabolic phenotype,
  - Refractory or severe hypoglycemia,
  - Abnormal response to therapy related to an underlying medical condition affecting vital organs or bodily systems,
  - Severe hypotonia,
  - Refractory seizures,
  - A high-risk stratification on evaluation for a Brief Resolved Unexplained Event with any of the following:
    i. Recurrent events without respiratory infection,
    ii. Recurrent witnessed seizure-like events, or
    iii. Required cardiopulmonary resuscitation,
  - Abnormal chemistry levels suggestive of inborn error of metabolism,
  - Abnormal cardiac diagnostic testing results suggestive of possible channelopathies, arrhythmias, cardiomyopathies, myocarditis, or structural heart disease, or
  - Family genetic history related to beneficiary’s condition.

## Future prospects for URGS

URGS has evolved rapidly in the twelve years since its initial description. Several recent advances have the potential to increase the diagnostic and clinical utility of URGS in addition to reducing cost and turnaround time.

## New URGS platforms and innovations

Paired, short-read (100 or 150 nucleotides (nt)) RGS and URGS (PSRGS), using sequencing-by-synthesis (SBS, Illumina) has been the mainstay technology for diagnostic URGS worldwide since initial description in 2012^7^. SBS was initially performed on the HiSeq 2500 in rapid run mode, followed by SP, S1, and S2 flowcells on the NovaSeq 6000 instrument with fastest time of 13.5 h (Fig. 2)^7,9,12,14,40^. During the last twelve months, however, the landscape of URGS technology has been transformed. In SBS, basecalling occurs after the URGS run ends (~20 h). In contrast, URGS with the Oxford Nanopore Promethion calls bases “live” during runs, as DNA molecules pass through nanopores at a speed of 400 nt/sec. By performing URGS on 48 Promethion flowcells simultaneously, diagnostic URGS can be performed in 7 h (Fig. 2)^41,42,125,126^. The most recent Illumina SBS platform, the NovaSeq X Plus, together with new SBS chemistry and 10B flowcells enables 20 URGS per 24-h run at a singleton, 40X consumable cost of $364 per individual. At the same time, Complete Genomics introduced the DNBSEQ-T7 instrument that enables 48 40X URGS in 24 h at a consumable cost of $200 and with similar sequence quality as SBS. New genome sequencing platforms from Ultima Genomics, Singular Genomics, and Element Biosciences have similar potential to reduce the cost or improve the scalability of URGS. For example, the Element Biosciences AVITI platform enables two 40X RGS in 38 h at a consumable cost of $240.

New bioinformatics pipelines and reference human genome sequences also have the potential to increase the diagnostic yield of URGS. The new human reference GRCh38 corrects ~1.5% of SNV and ~2.0% of indels that are incorrectly identified in GRCh37 since they lie within complex, discordant regions (DISCREPs) containing segmental duplications, patch sequences, and 109 Mb of alternate haplotypes^127–129^. GRCh37 DISCREPS include eight genes that cause Mendelian diseases. A recent telomere-to-telomere reference genome, CHM13, has further quality improvements that reduce SNV, indel and SV errors in 22 Mb of GRC38 DISCREPS, including ~270 complex medically relevant genes^130–133^. The full potential of these reference genomes to increase diagnostic yield requires use of the latest generation of read alignment and variant identification software, such as the Illumina DRAGEN v4.2, which uses machine learning and graph theory to map reads to alternate haplotypes in GRCh38 that differ in minority populations^127,128^.

Another key technology development has been long-read (N_50_ of ~30 kb) genome sequencing (LRGS) by Oxford Nanopore and Pacific Biosciences, and pseudo-long read sequencing from Complete Genomics and Illumina^134,135^. PSRGS has five shortcomings. Firstly, it is rather insensitive for structural variants (SV F_1_ score ~78% for SBS)^14^. In addition to much greater sensitivity for SVs (F_1_ score ~96%), LRGS characterizes the exact nucleotide boundaries of SVs and deconvolutes complex SVs (those with multiple copies, mixed duplications and deletions, and inversions)^20,136^. In contrast, PSRGS is limited to categorization of SVs as duplications or deletions, and breakpoint identification is not always possible or accurate. Secondly, PSRGS cannot typically resolve variants in 273 medically relevant genes with pseudogenes, low complexity or repetitive regions, LINE1 or Alu elements, or high-GC content repeat expansions^20,136–138^. This is because short reads map ambiguously within such regions. In contrast, long reads map unambiguously because they bridge across such regions into unique sequences. Many of these difficult genes cause disorders that affect newborns and may contain pathogenic variants in currently unsolved cases. Examples include Gaucher disease [MIM: 608013, 230800, 230900, gene *GBA*], congenital adrenal hyperplasia due to 21-hydroxylase deficiency [MIM:201910, gene *CYP21A2*], spinal muscular atrophy [MIM:23300, gene *SMN1*], hemophilia A [MIM:306700, gene *F8*] intron 22 inversion, deletion SVs in α-thalassemia (--/-- and --/-α, [MIM:604131, genes *HBA1* and *HBA2*]) and Duchenne muscular dystrophy [MIM:310200, gene *DMD*], campomelic dysplasia (MIM: 114290, gene *SOX9*), Pseudohypoparathyroidism type Ib (MIM: 603233, gene *GNAS*), Desbuquois dysplasia 2 (MIM:615777, gene *XYLT1*), and Coffin Siris syndrome 1 (MIM: 135900, gene *ARID1B*)^20,136–138^. Thirdly, PSRGS does not allow phasing of potentially causative compound heterozygous variants in recessive diseases. Phasing requires PSRGS of parent-child trios or use of alternative confirmatory tests. LRGS natively resolves phase by generating long haplotypes. Fourthly, in the ~60 short tandem repeat (STR) expansion disorders, LRGS performs accurate allele-specific STR sizing and internal sequence determination, while PSRGS only identifies approximate size of STR expansions^139,140^. This is diagnostically relevant since interrupting motifs can stabilize STR alleles and protect against full expansion. STR expansion disorders include myotonic dystrophy 1 [MIM:160900, gene *DMPK*] and central hypoventilation syndrome 1 [MIM:209880], which are common in NICU infants.

Finally, LRGS distinguishes 5-methylcytosine (5mC) from cytosine natively, whereas PSRGS currently does not. 5mC is an epigenetic imprint that regulates gene expression. There are at least 12 congenital imprinting syndromes, several of which lead to NICU admission^141,142^. By SNV-based haplotyping and enumeration of 5mC and C-containing reads at differentially methylation regions (DMRs), LRGS can diagnose both congenital imprinting syndromes and pathogenic STR expansion disorders including Desbuquois dysplasia type 2 [MIM: 615777], Angelman syndrome [MIM:105830], Prader-Willi syndrome [MIM:176270], facioscapulohumeral muscular dystrophy 1 [MIM:1589000], and Russell-Silver syndrome 1 [MIM:180860] (Fig. 2)^44–46,126,137,143^. Similarly, LRGS can distinguish benign (B) and pathogenic (P) variants through detection in blood gDNA of 5mC episignatures (disease-specific sets of CpG dinucleotide methylation changes across the genome). These can upgrade VUS to P or downgrade VUS to B in neurodevelopmental disorders (NDDs), improving diagnostic yield. While 5mC episignatures have been developed for ~70 NDDs, they are currently based on bisulfite treatment of gDNA and microarray hybridization. As ‘episignatures’ are validated for specific NDDs using LRGS, this will be an additional diagnostic use of the 5mC detection capability of LRGS.

One shortcoming of Oxford Nanopore LRGS is imprecision in identification of homopolymer insertion-deletion nucleotide variants (indels). Whereas PSRGS has indel F1 scores of 99.7%, that of Oxford Nanopore LRGS is 87.8%. Accurate identification of indels is critical for URGS and RGS, since these variants often lead to frame shifts at translation that are disease-causing. Excluding homopolymer indels, however, Oxford Nanopore LRGS has an F1 score of 98.9%.

It should be noted that these new platforms and capabilities, while very exciting, remain limited to research studies. In contrast to paired, short read, URGS and RGS, they have not yet been validated for clinical use in CLIA/CAP accredited diagnostic laboratories.

## Increasing use of artificial intelligence (AI) in URGS

AI is gaining broad interest for medical interpretation in areas as diverse as nuclear medicine imaging, echocardiography, ultrasonography, retinal imaging, dermatology, and histopathology^144–153^. AI provides information collection, integration, analysis, and interpretation capabilities that are many orders of magnitude greater than the human brain. With appropriate training, AI applications can attain performance in specialized interpretive tasks that equals that of medical specialists with decades of experience. Furthermore, AI can provide greater standardization and reproducibility than human experts.

URGS is well suited for AI since inputs and outputs are digitized (genotypes, phenotypes, gene-disorder dyads, diagnostic findings), large training datasets are becoming available, and variant interpretation has been codified as Boolean operators^12,14,91,154,155^. The initial applications of AI in URGS – basecalling, alignment, and variant identification (primary and secondary analysis) – are now well validated and universally employed^12,14,91,154,155^. Further gains in these AI applications are anticipated, both to improve performance, particularly for newer sequencing platforms, and to incorporate pangenome references and genome assemblies^133,156,157^. Four recent additional applications of AI in URGS will be briefly reviewed. The first is Natural Language Processing (NLP) of Electronic Medical Records (EMR) to provide deep phenotypes to broaden the scope of diagnostic interpretation of URGS^12^. EMRs, when extracted in controlled vocabularies, provide digital phenotype information. Traditionally, diagnostic interpretation of URGS was limited to a few patient phenotypes that were manually entered at time of order as Human Phenotype Ontology terms. The depth of phenotype entry varied between physicians and phenotype choice was biased by physician assessment of their clinical importance and relevance to a working differential diagnosis. Such sparse phenotyping limited the scope of analysis and interpretation since primary findings are genotypes that map to disorders that partially match the entered phenotypes, and diagnostic test reports are necessarily incomplete with regard to secondary or incidental findings. In one recent study of URGS, NLP identified 27-fold more phenotypic features than manual entry, including 5 times as many phenotypes that matched the diagnosed disorder in affected children than the phenotypes used in manual interpretation^12^. We recently found that NLP increased the analytic performance of phenotype extraction fivefold (from an F_1_ score of 16% by manual entry to 75%). The full capabilities of NLP are only starting to be examined in URGS. NLP can also identify negated phenotypes (which is important for pathognomonic clinical features of disorders), quantitative phenotype modifiers (e.g. phenotype frequency or severity), and longitudinal dynamics of phenotypes (for assessment of causality). Furthermore, NLP can explore the context of phenotypes within the EMR, which can help disambiguate meaning. For example, it is important to distinguish phenotypes mentioned in past medical history from those mentioned in family history. In newborns it is important to distinguish maternal and infant phenotypes. In the future, NLP can be utilized together with Boolean operators or AI to optimize quantitative comparisons of the goodness of fit of observed and expected phenotypes specifically for URGS in critically ill inpatient children.

The second application of AI in URGS is to automate routine diagnostic interpretation^12,14,91,92,154,155,158–160^. Current manual interpretation software systems provide over twenty annotations for each variant. Several of these annotations are already based on machine learning tools. In addition, annotated variants (or variant diplotypes) are rank ordered by machine learning for predicted pathogenicity. Furthermore, the entered phenotypes are matched to those of all known genetic diseases using machine learning to yield a comprehensive, rank ordered differential diagnosis. AI-based diagnostic interpretation goes one step further, performing additional Bayesian inference to associate each diplotype with a diagnostic score. One such tool (GEM, Fabric Genomics) correctly ranked over 90% of the causal genes among the top or second candidate and prioritized for review a median of 3 candidate genes per NICU infant diagnosed with a genetic disease^91^. Another tool correctly selected the causal diplotype in 97% of manually analyzed positive cases^158^. The initial use of AI-based interpretation of URGS will be to minimize laboratory director manual effort in ~90% of cases by short-listing the top candidate diagnoses together with the supporting evidence. In such cases it is possible to reduce laboratory director effort about tenfold^14,40,91,92^. In the remaining outlier or edge cases, manual interpretation will continue to be required.

The true positive and negative predictive value (PPV, NPV) of URGS, RGS, and RES results have not yet been adequately studied. Such studies are technically difficult since they require long-term follow up of cases. In the future, studies by the Undiagnosed Diseases Network (UDN) and increasing use of other ‘omics platforms for functional confirmation of molecular diagnoses should be informative^161,162^. Prior studies by the UDN have not addressed this question since their experience predates broad use of URGS, RGS and RES. It will also be important to compare the PPV and NPV of manual and AI-assisted interpretation. One challenge the AI field has struggled with is gold standard comparisons. Prospective comparisons of AI and manual methods are particularly needed.

The third application of AI in URGS is clinical decision support for therapeutic interventions following return of a likely genetic disease diagnosis^14,163,164^. Currently management guidance requires subspecialist or superspecialist consultation, which invokes delays in implementation. This is particularly true for ultra-rare genetic diseases. A barrier to implementation of precision medicine for genetic diseases is that evidence for effectiveness of most therapeutic interventions is largely limited to case reports and small case series. As URGS becomes more widely used, results will increasingly be returned to hospitals lacking a full complement of pediatric subspecialists, genetic counselors, and to pediatricians who are unfamiliar with the diagnosed disorders, invoking increased delays. This gap in physician knowledge is also increasing because of a rapid increase in the number of investigational new drug applications and approvals for new gene-based therapeutics (Fig. 2)^165^. Recently several groups have described AI-assisted clinical decision support tools for therapeutic interventions for rare genetic diseases^14,165^.

Finally, AI can be used to automate the identification of patients who are likely to benefit from URGS, RGS, or RES. Machine learning, together with NLP of the EHR, has been used to develop classifiers that distinguish between NICU infants who have or have not historically received URGS, RGS, or RES^166^. As URGS, RGS, and RES are implemented in NICUs without prior experience, such tools will be important in achievement of high diagnostic rates. With appropriate training, generative AI can also be used to alleviate the burden of pre- and post-test genetic counseling^167,168^. Further work is needed to define best practices for genetic counseling in high acuity settings.

As noted for sequencing technology innovations, the clinical utility of these AI tools has not been broadly evaluated in prospective clinical studies. Considerable further comparative studies of AI and manual methods are needed. In summary, however, the use of AI has the potential to improve scalability and standardization of URGS and genome-informed care and to reduce cost, time-to-intervention, and geographic disparities^169^.

## Conclusion

There are ~10,000 currently known single locus (Mendelian) genetic diseases. Collectively they are a leading cause of hospitalization, ICU admission, mortality, and healthcare cost in infants and children. However, the true incidence of genetic diseases among infants and children admitted to intensive care units remains unknown. Initially described in 2012, URGS is a purpose-developed, clinical method for timely diagnosis of almost all genetic diseases in high acuity NICU, PICU and, CVICU patients. In 44 studies of rapid genomic sequencing, 37% of children in ICUs with diseases of unknown etiology or with suspected genetic disorders received a genetic disease diagnosis. In 39 clinical utility studies, rapid genomic sequencing changed management in 26% of children tested. In nine cost effectiveness studies, rapid genome sequencing led to $14,265 in net cost savings per child tested. URGS outperformed rapid genome or exome sequencing in these studies with 49% rate of diagnosis and 37% clinical utility. These metrics are expected to improve as URGS methods evolve, test costs decrease, and testing is implemented within precision medicine delivery systems attuned to the needs of neonatologists and intensivists. The evidence for clinical utility and cost effectiveness is adequate to conclude that diagnostic URGS should be considered in all infants and children with diseases of uncertain etiology at time of ICU admission. Clinical URGS is currently performed in less than 5% of the ~200,000 infants and children who fit the enrollment criteria of these research studies. The major barriers to broad implementation of diagnostic URGS are under-recognition of the possibility of genetic diseases by physicians, limited or absent reimbursement, inpatient barriers to test ordering, and current formatting of URGS as a test rather than within a rapid precision medicine delivery system attuned to the needs of hospital physicians, such as neonatologists and intensivists. Little implementation science has yet been done to optimize the clinical utility or outcomes associated with URGS. The gap between actual and optimal outcomes in critically ill children with genetic diseases is currently increasing since growth in testing lags growth in the number of children who would benefit because of newly approved therapies for these diseases. In five years, it is anticipated that there will be effective treatments for at least 1000 genetic diseases and diagnosis by URGS will be available within one day nationwide at a cost of $2500. It is also anticipated that URGS has the potential to reduce childhood mortality in the US significantly within 5 years.

The true incidence of genetic diseases among infants and children admitted to ICUs remains unclear. Therefore, the optimal breadth of use of URGS is uncertain. For example, there is a need for clinical utility and cost effectiveness studies of URGS in community hospital ICUs, in general beds in children’s hospitals, and in critically ill children who are not suspected of having a genetic disease.

The maximal diagnostic yield and clinical utility of URGS are uncertain. Technical advances in URGS, some of which were reviewed here, have the potential to double the rate of diagnosis. A significant unknown is the added value of combining URGS with equally rapid functional ‘omic assays, such as transcriptome sequencing, metabolomics, and proteomics using readily available biological samples. As our knowledge of variant pathogenicity increases it is anticipated that the frustrating term variant of uncertain significance will become a historical artifact.

Little work has yet been done to optimize the clinical utility or outcomes associated with URGS. There is considerable need for implementation science and process engineering to transform testing into generalizable rapid precision medicine delivery systems. Significant gains in the rate of change in management following URGS are possible.

Long term trends suggest that in five years there will be effective treatments for at least 1,000 genetic diseases and that diagnosis by URGS will be available within a day nationwide at a cost of ~$2,500 (Fig. 2). In five years there is the potential for infant and childhood mortality in the US and UK to have been reduced by several percent through use of URGS as a first-tier, standard of care test for children in ICUs with diseases of uncertain etiology.

## Data Availability

This is a review article. There is no associated primary data.
